# Complement Effectors of Inflammation in Cystic Fibrosis Lung Fluid Correlate with Clinical Measures of Disease

**DOI:** 10.1371/journal.pone.0144723

**Published:** 2015-12-07

**Authors:** Laura A. Sass, Pamela S. Hair, Amy M. Perkins, Tushar A. Shah, Neel K. Krishna, Kenji M. Cunnion

**Affiliations:** 1 Department of Pediatrics, Eastern Virginia Medical School, 700 West Olney Road, Norfolk, Virginia, United States of America; 2 Children's Specialty Group, 811 Redgate Avenue, Norfolk, Virginia, United States of America; 3 Children’s Hospital of The King’s Daughters, 601 Children’s Lane, Norfolk, Virginia, United States of America; 4 Department of Microbiology and Molecular Cell Biology, Eastern Virginia Medical School, 700 West Olney Road, Norfolk, Virginia, United States of America; University of Leicester, UNITED KINGDOM

## Abstract

In cystic fibrosis (CF), lung damage is mediated by a cycle of obstruction, infection, and inflammation. Here we explored complement inflammatory effectors in CF lung fluid. In this study soluble fractions (sols) from sputum samples of 15 CF patients were assayed for complement effectors and analyzed with clinical measurements. The pro-inflammatory peptide C5a was increased 4.8-fold (P = 0.04) in CF sols compared with controls. Incubation of CF sols with *P*. *aeruginosa* or *S*. *aureus* increased C5a concentration 2.3-fold (P = 0.02). A peptide inhibitor of complement C1 (PIC1) completely blocked the increase in C5a concentration from *P*. *aeruginosa* in CF sol *in vitro* (P = 0.001). C5a concentration in CF sol correlated inversely with body mass index (BMI) percentile in children (r = -0.77, P = 0.04). C3a, which has anti-inflammatory effects, correlated positively with FEV1% predicted (r_s_ = 0.63, P = 0.02). These results suggest that complement effectors may significantly impact inflammation in CF lung fluid.

## Introduction

Cystic fibrosis (CF) afflicts 30,000 individuals in the United States with respiratory failure causing the majority of deaths. Progressive destruction of lung parenchyma is mediated by a cycle of obstruction, infection with bacterial pathogens, and inflammation [1]. As the cycle repeats, lung damage progresses to lung scarring and finally pulmonary failure.

**Fig 1:**
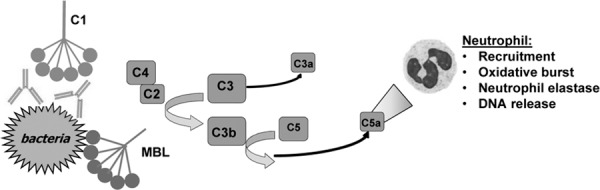
Bacteria initiating classical (C1) or lectin (MBL) pathway complement activation with C5a-mediated neutrophil recruitment and activation.

The most destructive inflammatory cascade in the human body is the complement system, which contributes to host tissue damage in numerous inflammatory disease processes [2]. Recent evidence shows complement proteins are major constituents of lung fluid in CF, where C3 and C4 account for two of the four most prevalent proteins [3]. Thus, complement may play a larger role in CF lung inflammation than previously suspected. Antibody binding to bacteria can activate the classical complement pathway via the initiating component C1 (**Fig 1**). Another serum protein, mannose-binding lectin (MBL), can directly bind foreign sugars on the surface of pathogenic bacteria activating the lectin complement pathway. The classical and lectin pathways proceed via C4 leading to downstream activation of C3 [4]. C3 activation generates the complement effector C3a and covalently binds cells with the opsonins C3b and iC3b. C3b initiates activation of C5, generating the extremely potent anaphylatoxin C5a. C5a is among the most powerful stimulants for neutrophil migration and activation, leading to oxidative burst and degranulation [4,5]. Neutrophil death following degranulation is a major source of the viscous DNA contributing to airway obstruction [6,7]. Neutrophil granules release neutrophil elastase, a major contributor to lung damage [8–10]. Additional properties of C5a that may also contribute to CF lung disease are stimulation of histamine release, enhancement of vascular permeability, and smooth muscle contraction [4]. The known inflammatory properties of C5a are consistent with the increasing evidence of the role of C5a in inflammatory lung diseases [11,12], including acute lung injury [12]. Thus, multiple lines of reasoning suggest that complement modulation of inflammation may be a major contributor to lung damage in CF.

Significant investigation has focused on the interactions of leading CF pathogens *P*. *aeruginosa* and *S*. *aureus* with the complement system. The work of several investigators has been summarized in a meta-analysis suggesting that MBL insufficiency is associated with earlier acquisition of *P*. *aeruginosa*, reduced pulmonary function, and earlier death or lung transplantation [13]. High levels of specific IgG3 plasma antibodies against *P*. *aeruginosa* are associated with higher complement-activation capacity and poor lung function as assessed by vital capacity [14]. Antibodies against neutral polysaccharides on *P*. *aeruginosa* can increase C3 opsonization for certain strains [15], but do not protect against infection likely secondary to O side-chains and alginate interfering with opsonic killing [16]. CFTR has been demonstrated on blood monocytes and is associated with reduced complement-dependent opsonization of *P*. *aeruginosa* [17]. Although a great deal of investigation has focused on *S*. *aureus* control of complement activation and evasion of complement effectors [18,19], very little has been done in the context of cystic fibrosis. In summary, many studies have focused on the many mechanisms these pathogens utilize to evade complement-mediated immune mechanisms with a few studies hinting at the potential role of complement in CF lung damage.

A modicum of investigation into the potentially important role of C5a in the CF lung was performed nearly 30 years ago. In 1986, Fick et al [20] described the presence of increased amounts of C5a, measured by radioimmunoassay, in the bronchoalveolar lavage (BAL) of 9 CF patients with stable lung disease compared with BAL from healthy controls. The CF BAL fluids were chemotactic for neutrophils, correlating with C5a concentrations. Two (2) CF patients with the lowest C5a measurements were noted to have normal FEV_1_ and FVC measurements, suggesting a potential association with lung damage. To our knowledge no further studies have been performed to test whether C5a concentrations in CF lung fluid correlate with lung disease in CF. The experiments described here evaluate complement activation and effectors in CF sputum and provide preliminary data correlating complement effectors with clinical measures of disease.

## Results

### Complement anaphylatoxins in CF lung fluid

**Fig 2:**
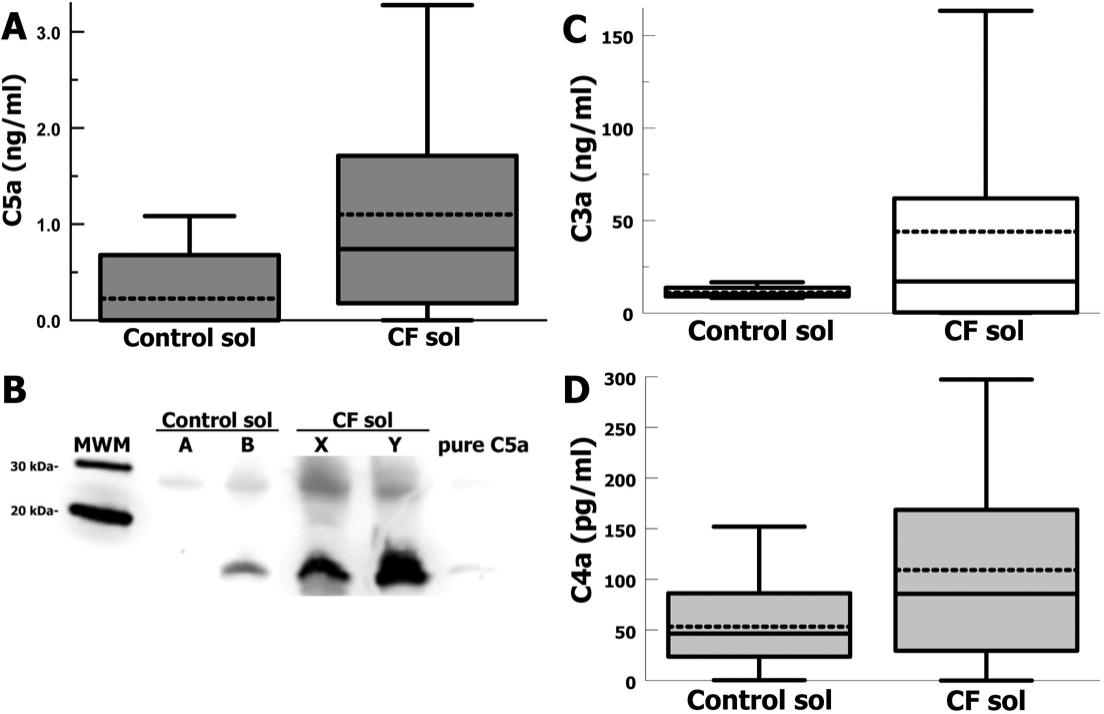
Complement anaphylatoxins in CF and control lung fluid. (A) C5a concentrations in sol fractions from sputum of CF patients (n = 15) and controls (n = 3). Box shows quartiles, whiskers are 90^th^ and 10^th^ percentile, and dashed line is the mean. P = 0.04. (B) C5a Western blot for sputum sols for 2 healthy controls (A and B) and 2 CF subjects (X and Y). (C) C3a concentrations in sol fractions from sputum of CF patients (n = 14) and controls (n = 4). Box shows quartiles, whiskers are 90^th^ and 10^th^ percentile, and dashed line is the mean. P = 0.03. (D) C4a concentrations in sol fractions from sputum of CF patients (n = 15) and controls (n = 5). Box shows quartiles, whiskers are 90^th^ and 10^th^ percentile, and dashed line is the mean. P = 0.05.

In order to evaluate whether complement anaphylatoxins were elevated in CF lung fluid we assayed CF sols from the sputum of CF patients for each complement anaphylatoxin and compared these results with sputum sols from healthy human controls. The most inflammatory complement anaphylatoxin is C5a, which we assayed by ELISA and Western blot. Mean C5a concentration in CF sols was 4.8-fold higher (*p* = 0.04) compared with the mean for healthy controls (**Fig 2A**). Qualitative analysis by Western blot probing for C5a confirmed that large amounts of C5a are present in CF sols compared with controls (**Fig 2B**). C3a is a complement effector that is generated during activation of the central complement component C3. Mean C3a concentrations in CF sols was 4-fold higher (*p* = 0.03) compared with controls (**Fig 2C**). C4a is the least potent of complement anaphylatoxins and is generated during classical or lectin pathway complement activation. Mean C4a concentration was 2-fold higher in CF sols (*p* = 0.05) compared with controls (**Fig 2D**). Together these data show that the concentration of complement anaphylatoxins in CF lung fluid is significantly elevated, suggesting significant complement activation in CF lung fluid. The potent ability of C5a to recruit neutrophils and stimulate degranulation [4,21,22], suggests that C5a could contribute to the high concentrations of neutrophil elastase in CF lung fluid, which is associated with parenchymal destruction. Additionally, the elevated levels of C4a in CF lung fluid, suggests that much of the complement activation occurring in CF lung fluid may be occurring via the classical or lectin complement pathways.

### Complement opsonization of *S*. *aureus* in CF lung fluid

**Fig 3:**
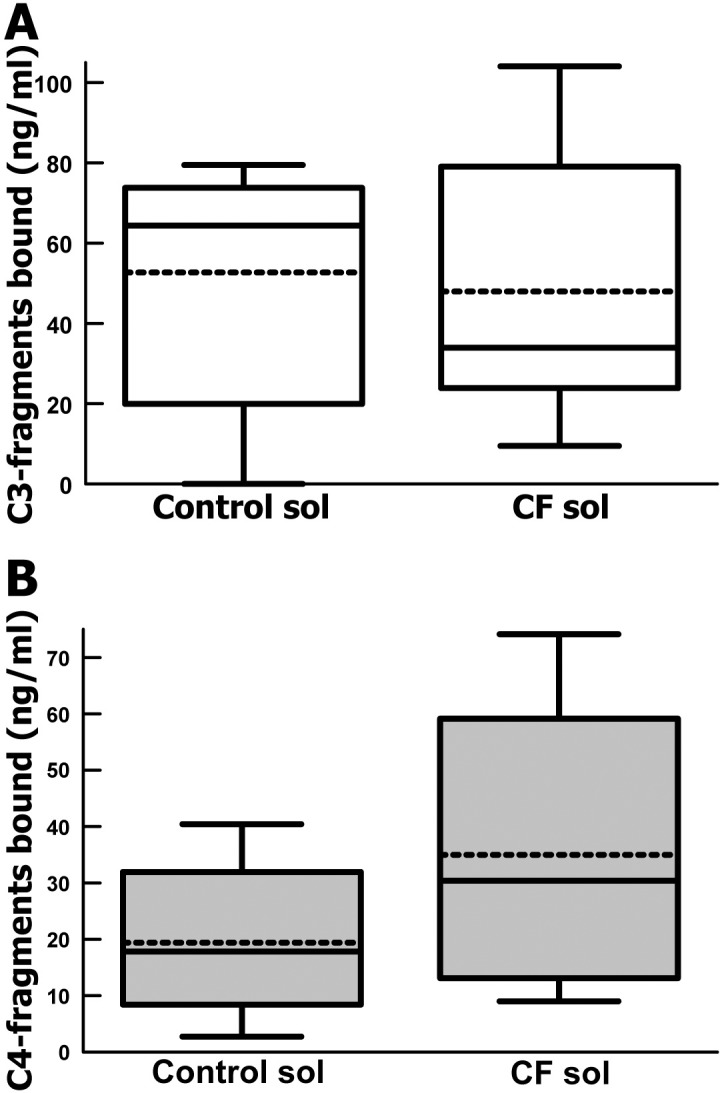
Complement opsonization of *S*. *aureus*. (A) *S*. *aureus*-bound C3-fragments after incubation in sol fractions from sputum of CF patients (n = 5) and controls (n = 3). Box shows quartiles, whiskers are 90^th^ percentile, and dashed line is the mean. P = 0.42. (B) *S*. *aureus*-bound C4-fragments after incubation in sol fractions from sputum of CF patients (n = 5) and controls (n = 3). Box shows quartiles, whiskers are 90^th^ and 10^th^ percentile, and dashed line is the mean. P = 0.13.

In order to evaluate whether complement in CF lung fluid could adequately opsonize a pathogenic bacteria, we evaluated CF sol opsonization of *S*. *aureus*. Given the large amounts of complement activation that had already occurred in the CF lung fluids, it was important to determine whether there was residual complement-mediated host defense. *S*. *aureus* were incubated with CF or control sols, washed and stripped of bound C3-fragments and bound C4-fragments. *S*. *aureus* was robustly opsonized in CF sol yielding a nearly identical mean level of bound C3-fragments compared with normal controls (**Fig 3A**). *S*. *aureus* was also robustly bound by C4-fragments (*p* = 0.13) with a non-significant trend towards increased mean C4-fragment binding by CF sols compared with normal controls (**Fig 3B**). These results suggest that CF lung fluid retains a normal capacity to opsonize bacteria, suggesting that this facet of host defenses is not compromised. These results also show that despite significant complement activation having occurred, as evident by very high anaphylatoxin levels, significant activable complement remains in CF lung fluid. This suggests a cycle of complement activation and repletion consistent with persistent inflammation. The robust opsonization with C4-fragments suggests that the classical or lectin complement pathway is active in CF lung fluid and may be the predominant pathway of complement activation.

### C5a generation in CF lung fluid by *P*. *aeruginosa* and *S*. *aureus*

**Fig 4:**
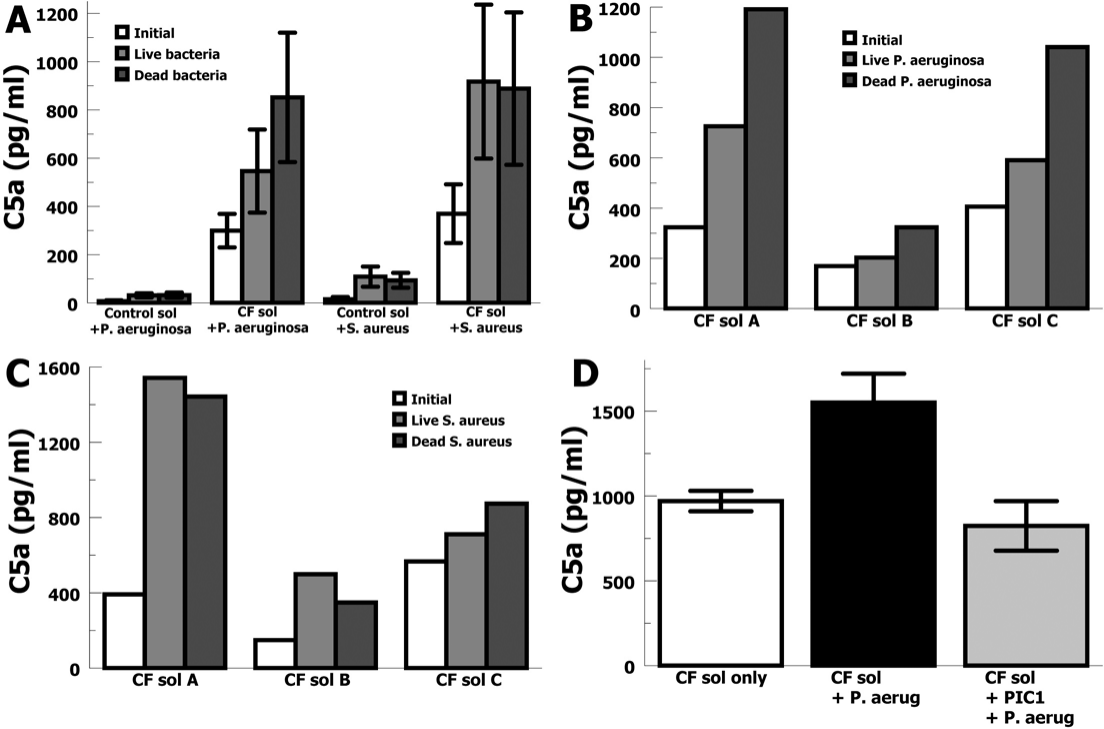
C5a generated by bacteria in CF sols. (A) C5a concentrations in sol fractions from sputum of CF patients (n = 3) or controls (n = 3) before and after incubation with live or dead *P*. *aeruginosa* or *S*. *aureus*. Data are means ± SE. C5a was generated in CF sol in the presence of *P*. *aeruginosa* (P = 0.03) or *S*. *aureus* (P = 0.03). (B) C5a concentrations in sol fractions from sputum of CF patients (subjects A, B, and C) before (Initial) and after incubation with live or dead *P*. *aeruginosa*. (C) C5a concentrations in sol fractions from sputum of CF patients (subjects A, B, and C) before (Initial) and after incubation with live or dead *S*. *aureus*. *(*D) C5a concentrations in CF sols that were incubated alone in buffer (CF sol only), incubated with dead *P*. *aeruginosa* (CF sol + P. aerug), or incubated with a classical/lectin complement pathway inhibitor and dead *P*. *aeruginosa* (CF sol + PIC1 + P. aerug).

In order to evaluate whether CF lung fluid challenged with pathogenic bacteria commonly present in CF lungs would generate new C5a, the most inflammatory anaphylatoxin, we incubated CF sols with live and dead *P*. *aeruginosa* and *S*. *aureus*. We tested live and dead versions of each bacterium because both forms are likely to be present in an infected CF lung. Additionally, we wanted to determine if secreted factors or adaptive changes that could be produced by live bacteria would alter C5a generation. C5a concentrations were measured in CF and control sols prior to incubation with bacteria and then afterwards to determine new C5a generation. Incubation of CF sols with live or dead *P*. *aeruginosa* lead to an average increase in C5a generation of 2.3-fold (*p* = 0.02) and similarly incubation with live or dead S. aureus led to an average increase in C5a generation of 2.4-fold (*p* = 0.02) (**Fig 4A**). The increases in C5a generation for the control sols was statistically significant, but much less than was found for the CF sols in terms of absolute amounts of C5a generated. These data also showed a difference in C5a generation between live or dead *P*. *aeruginosa* (*p* = 0.05), but not between live or dead *S*. *aureus*. This relationship between live or dead *P*. *aeruginosa* challenge appeared consistent for the different CF sol samples (**Fig 4B**), but there was no consistent relationship between live or dead *S*. *aureus* challenge (**Fig 4C**). These data show that *P*. *aeruginosa* and *S*. *aureus* both provoke robust generation of the highly inflammatory C5a anaphylatoxin in CF lung fluid, suggesting that the presence of these bacteria in the CF lung may be enhancing inflammation and subsequent host tissue damage via this mechanism. These data also suggest that live *P*. *aeruginosa* may have some ability to moderate C5a generation in CF lung fluid compared to dead *P*. *aeruginosa*.

The experiments above show that in CF lung fluid complement activation is occurring to generate complement effectors, yet more complement activation is possible suggesting that complement components are also being repleted. In order to evaluate the likely complement pathways by which *P*. *aeruginosa* was activating C5a generation, we tested an inhibitor of classical and lectin complement pathway activation. Peptide inhibitor of complement C1 (PIC1) is a small peptide inhibitor of the classical and lectin pathways of complement activation which binds C1q or MBL to prevent cognate serine proteases from cleaving C4 [23–26]. CF sols were incubated in buffer alone, with dead *P*. *aeruginosa*, or with PIC1 and dead *P*. *aeruginosa* (**Fig 4D**). Dead *P*. *aeruginosa* increased C5a concentration by 1.6-fold compared to incubation of the CF sol in buffer alone. Addition of PIC1 to the CF sol decreased C5a generation by *P*. *aeruginosa* (*p* = 0.001) to a level not significantly different from CF sol alone (*p* = 0.22). Thus, addition of a classical/lectin pathway inhibitor blocked C5a generation by *P*. *aeruginosa*, suggesting that *P*. *aeruginosa* activates complement and C5a generation via the classical or lectin complement pathways. This finding is congruent with elevated C4a levels and robust C4-fragment opsonization and together they support a major role for classical/lectin pathway activation in CF lung fluid.

### Complement anaphylatoxin correlation with clinical characteristics

**Fig 5:**
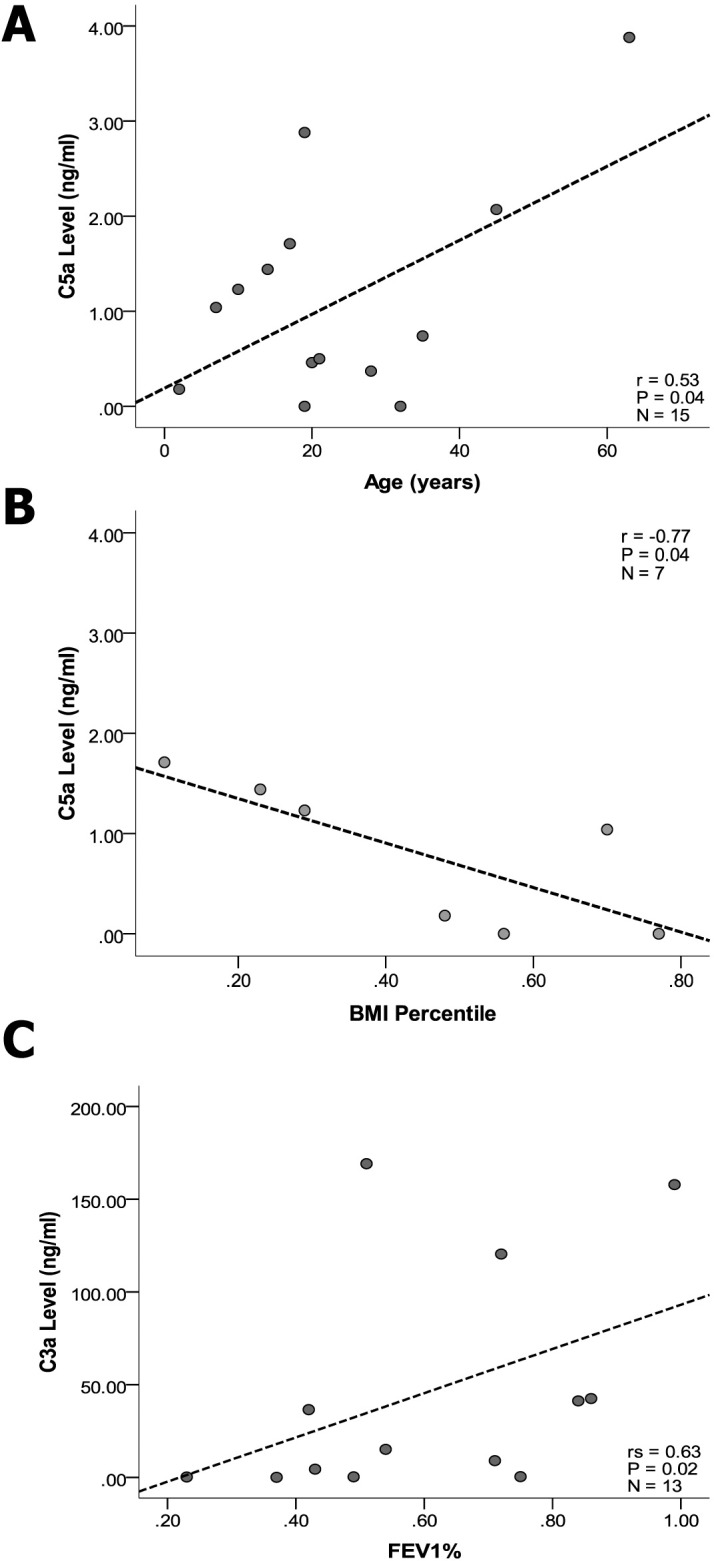
Correlation plots for complement effectors and clinical measures. (A) C5a concentrations in CF sols positively correlate with increasing age, r = 0.53, *p* = 0.04. (B) C5a concentrations in CF sols correlate inversely with BMI percentile in children, r = -0.77, *p* = 0.04. (C) C3a concentrations in CF sols positively correlate with FEV1%, r_s_ = 0.63, *p* = 0.02.

Because this is a pilot study to demonstrate proof of concept, the numbers of CF sputum samples was small, n = 15. Clinical data was collected with each sputum sample and we evaluated whether, despite the small sample size, any trends with clinical measures were identifiable. The clinical characteristics for these 15 subjects are shown in **S1 Table**. The subjects span a wide range of ages from 2–65 years old with a median age of 19. Median FEV1% predicted was 59; for children (n = 7) median BMI was 48% and for adults (n = 4) median BMI was 23.87 mg/kg^2^. Clinical data collected at the time of sputum sampling was obtained for a wide range of measures including FEV1% predicted, BMI percentage (children), bronchiectasis, CFRD status, pathogenic microorganisms cultured from the sputum and medications (i.e. corticosteroids, azithromycin, and antibiotics). Statistical correlations were assessed between the anaphylatoxins, C5a and C3a, and the clinical measures, see **S2 Table**. Increased concentration of the highly inflammatory C5a positively correlated with increased age (r = 0.53, *p* = 0.04), as shown in **Fig 5A**. Increased C5a concentration correlated inversely with BMI percentile in children (r = -0.77, *p* = 0.04) as shown in **Fig 5B**. Increased C3a levels positively correlated with increased FEV1% predicted (r_s_ = 0.63, *p* = 0.02), as shown in **Fig 5C**. Microorganisms (i.e. *P*. *aeruginosa*, *B*. *cepacia*, *S*. *aureus*, or yeast) recovered from the sputum, corticosteroids inhaled or systemic, azithromycin, or antibiotics inhaled or systemic, did not show correlation with C5a or C3a level. Despite the small numbers of samples, these results show that increasing C5a concentration correlated with decreased BMI percentile in children suggesting that increased complement inflammatory C5a in lung fluid may be associated with poorer overall health in children with CF. C3a level positively correlated with FEV1% predicted, suggesting a potentially protective effect from C3a on CF lung function.

## Discussion

These data confirm the findings of Fick et al [20] that CF lung fluid contains increased levels of anaphylatoxin C5a. Our data expand upon these findings by showing that sputum samples can be used to measure C5a and other complement effectors in CF lung fluid. The ability to use sputum samples allows for much larger longitudinal studies to be done in the future to further elucidate the role of complement effectors in the CF lung.

Our data show that despite high levels of activated anaphylatoxins present in CF sputum, considerable quantities of activable complement are available to opsonize bacteria. We further show that large amounts of new C5a are generated by CF sols when they are incubated with *P*. *aeruginosa* or *S*. *aureus*. These results suggest that there is a continuous cycle of complement activation and repletion in CF lung fluid consistent with the cycle of infection and inflammation, which is felt to play a major role in CF lung damage. Additionally, we show that the generation of C5a by *P*. *aeruginosa* can be blocked with a classical/lectin pathway inhibitor.

Given the small numbers of CF sputum samples tested, no strong conclusions about associations with clinical measures can be made with confidence. BMI in children with CF is multifactorial in nature, but generally regarded as a measure of overall health (i.e. low BMI is an independent predictor of mortality). Thus, elevated C5a concentration in the lung may be associated with poorer overall health in children with CF. The association of high C5a with low BMI percentile is from a small data set and needs to be validated in a larger study, but could potentially translate clinically as a biomarker for risk of wasting in children with CF.

Interestingly, elevated sputum C3a levels correlated positively with higher FEV1% predicted measurements. C3a has been shown to have anti-inflammatory effects in ischemia-reperfusion and septicemia by inhibiting neutrophil mobilization [27]. Thus, C3a may have protective effects in cystic fibrosis by regulating neutrophil activity [28]. Alternatively, C3a could be a marker of robust complement activation and activity against bacterial pathogens in the CF lung. However, the considerable literature describing multiple complement-evasion strategies for *P*. *aeruginosa* and *S*. *aureus* [19,29–34], suggest this is unlikely. These strategies include AprA degradation of C2 [31], Tuf recruitment of factor H [35], elastase degradation of C3 [36], and protease IV degradation of C1q and C3 [37]. Clearly, this association deserves further elucidation in a larger clinical study to determine whether this correlation is upheld and whether the C3a level trends with FEV1% predicted over time.

It remains unclear the extent to which complement is effective against bacterial pathogens in the CF lung; clearly normal host defenses are altered to such an extent that eradication of bacterial pathogens (e.g. *P*. *aeruginosa*, *B*. *cepacia* and MRSA) rarely occurs. It is plausible that complement may be modulating inflammation in the CF lung, but providing little antimicrobial effect. Thus, there may be an opportunity in the future to pharmacologically modulate complement effectors in CF lung fluid without further impairing already poor immunological control of pathogens.

## Materials and Methods

### Ethics Statement

Sputum samples were obtained from consented patients as part of their standard of care visit at the Children’s Hospital of The King’s Daughters Cystic Fibrosis Center under an Eastern Virginia Medical School IRB approved protocol 12-08-EX-0200. Written consent was obtained. The EVMS IRB specifically approved this study under protocol 12-08-EX-0200. Control sputum samples were obtained from healthy human volunteers.

### Sputum sols

Expectorated sputum samples were placed immediately on ice. The soluble (sol) fraction was generated by cold (4°C) centrifugation at 14,000 g for 60 minutes and recovery of the free flowing liquid fraction, similar to methods previously described [38]. Sol fractions were non-viscous and not normalized for protein content, consistent with previously described methods [39].

### Clinical data

Clinical data were obtained from data entered into Port CF for the clinic visit at which the sputum sample was collected and from review of the medical record. The FEV1% predicted was performed the same day the sputum was collected. Bronchiectasis was scored based on the most recent radiographic: 0 = normal; 1 = 1 lobe, mild; 2 = 2–4 lobes; 3 = all lobes. Cystic fibrosis related diabetes (CFRD) status was based on the most recent endocrinology assessment prior to obtaining the sputum sample. CFRD status was scored: 0 = normal; 1 = glucose intolerance; 2 = CFRD. Organisms were recorded from routine culture performed in the clinical microbiology laboratory. The organisms were categorized as to whether the following were present or absent: *P*. *aeruginosa*, *S*. *aureus*, *B*. *cepacia* complex, or *Candida* species. Patient medications at the time of clinic visit were categorized as to whether the following were present or absent: systemic corticosteroid, inhaled corticosteroid, azithomycin, inhaled antibiotic, or systemic antibiotic (excluding azithromycin).

### ELISAs

The C5a, C3a, and C4a concentrations in sputum sols were measured via ELISA kit (R&D Systems, Minneapolis, MN or BD Biosciences, San Jose, CA) [40–42]. Bound C3-fragments were measured using a total C3 ELISA using a goat anti-human C3 antibody (Complement Technology, Tyler, TX) for capture and a chicken anti-human C3 antibody (Sigma, St. Louis, MO) for detection, as previously described [43]. Bound C4-fragments were evaluated via same ELISA using a goat anti-human C4 antibody for capture and a chicken anti-human C4 antibody (Abcam, Cambridge, MA) for detection.

### Western blots

C5a fragments were analyzed by Western Blot using a mouse anti-human C5a antibody (R&D Systems) to probe followed by a goat anti-mouse HRP antibody (Sigma) and detected with ECL.

### *S*. *aureus* opsonization with CF sols

*S*. *aureus* strain Reynolds was grown in 2% NaCl Columbia broth at 37°C to log phase, washed twice and resuspended to 1 × 10^9^ cells/ml in GVBS^++^ buffer. An equal volume of bacteria and sol were incubated for one hour at 37°C. The bacteria were washed twice with GVBS^-—^buffer and then stripped of bound complement fragments using methylamine, as previously described [44].

### C5a generated in CF sols by live and dead *P*. *aeruginosa* and *S*. *aureus*

*Pseudomonas aeruginosa* and *S*. *aureus* were grown in broth to log phase and washed. Both bacteria were gently heat killed by incubating in a 70°C water bath for 15 min. Sols were incubated with live or dead *P*. *aeruginosa* or *S*. *aureus* at equal volumes for one hour at 37°C. Samples were sedimented and the supernatant recovered. Equal volume of CF sol and PIC1, a PEGylated derivative of polar assortant (PA) [23,24,26], at 50 mg/ml, or saline, was combined for 30 minutes before adding 5 × 10^7^ CFU heat-killed *P*. *aeruginosa* for 30 minutes. Samples were sedimented and the supernatant recovered.

### Statistical analysis of clinical measures with C5a/C3a

The data were analyzed using SAS 9.4 (SAS Institute, Cary, NC) and SPSS 19 (SPSS Inc., Chicago, IL) software. The level of significance was set at 0.05 and all hypothesis tests were two-sided. Pearson and Spearman correlation coefficients were reported, where appropriate, for C5a level, C3a level, and the clinical measures. Descriptive statistics (mean, median, and interquartile range (IQR)) were reported for C5a and C3a level stratified by clinical measures. Simple linear regression and Mann-Whitney *U* tests were used, where appropriate, to determine associations between C5a level, C3a level, and the clinical measures. A multivariable linear regression model for FEV1% was used to determine associations with C5a level and C3a level.

### Statistical analysis of laboratory data

Medians, quartiles, and 90^th^ percentiles were calculated using PSI plot. Means and standard error of the means (SEM) were calculated from independent experiments. Statistical comparisons were made using Student’s t-test where appropriate.

## Supporting Information

S1 Table Characteristics of the CF subjects. (DOCX)

S2 Table Correlation coefficients for complement effectors and clinical measures. (DOCX)

## Abbreviations

BAL: Bronchoalveolar Lavage
BMI: Body Mass Index
CF: Cystic Fibrosis
CFRD: Cystic Fibrosis Related Diabetes
FEV1%predicted: Forced Expiratory Volume in 1 second, percent of predicted
PIC1: Peptide Inhibitor of Complement C1
MRSA: Methicillin-resistant Staphylococcus aureus
