# Supplementary material for: Complement Effectors of Inflammation in Cystic Fibrosis Lung Fluid Correlate with Clinical Measures of Disease
Source: PLoS One. 2015 Dec 7;10(12):e0144723. doi: 10.1371/journal.pone.0144723 (PMC4671727; doi:10.1371/journal.pone.0144723)
Supplement: S1 Table — (DOCX) [file pone.0144723.s001.docx]

| **S1 Table**. Characteristics of the CF subjects. | |
| --- | --- |
|  | **Median**  **(range)** |
| **Age, y** | 19  (2 – 65) |
| **Gender, % female** | 60 |
| **Child BMI, %** | 26  (10 – 70) |
| **Adult BMI** | 23.8  (21.8 – 25.9) |
| **FEV1%** | 59  (23 – 99) |
