# Supplementary material for: Complement Effectors of Inflammation in Cystic Fibrosis Lung Fluid Correlate with Clinical Measures of Disease
Source: PLoS One. 2015 Dec 7;10(12):e0144723. doi: 10.1371/journal.pone.0144723 (PMC4671727; doi:10.1371/journal.pone.0144723)
Supplement: S2 Table — (DOCX) [file pone.0144723.s002.docx]

| **S2 Table.** Correlation coefficients for complement effectors and clinical measures | | |
| --- | --- | --- |
| **Correlation coefficient**  **P value (H_0_: rho=0)**  **N** | **C5a (ng/ml)** | **C3a (ng/ml)** |
| **Age, y** | 0.53^a^  0.04  15 | -0.12^b^  0.68  14 |
| **Child BMI, %** | -0.77^a^  0.04  7 | -0.39^b^  0.38  7 |
| **FEV1%** | 0.04^a^  0.89  14 | 0.63^b^  0.02  13 |
| **Bronchiectasis Score** | 0.12^b^  0.68  15 | 0.15^b^  0.61  14 |
| **CFRD Score** | 0.27^b^  0.38  13 | 0.01^b^  0.98  12 |
| Bronchiectasis score: 0 = normal; 1 = 1 lobe, mild; 2 = 2-4 lobes; 3 = all lobes  CFRD score: 0 = normal; 1 = glucose intolerance; 2 = CFRD  ^a^ Pearson correlation coefficient  ^b^ Spearman correlation coefficient | | |
